# Skin Microbiome and Treatment-Related Skin Toxicities in Patients With Cancer: A Mini-Review

**DOI:** 10.3389/fonc.2022.924849

**Published:** 2022-07-15

**Authors:** Brianna N. Richardson, Jolinta Lin, Zachary S. Buchwald, Jinbing Bai

**Affiliations:** ^1^ Emory College, Emory University, Atlanta, GA, United States; ^2^ Department of Radiation Oncology, Winship Cancer Institute, School of Medicine, Emory University, Atlanta, GA, United States; ^3^ Nell Hodgson Woodruff School of Nursing, Emory University, Atlanta, GA, United States

**Keywords:** cancer, chemotherapy, immunotherapy, radiation therapy, skin microbiome, adverse event, skin toxicity, 16S rRNA

## Abstract

The human skin hosts millions of bacteria, fungi, archaea, and viruses. These skin microbes play a crucial role in human immunological and physiological functions, as well as the development of skin diseases, including cancer when the balance between skin commensals and pathogens is interrupted. Due to the linkages between inflammation processes and skin microbes, and viral links to skin cancer, new theories have supported the role a dysbiotic skin microbiome plays in the development of cancer and cancer treatment-related skin toxicities. This review focuses on the skin microbiome and its role in cancer treatment-related skin toxicities, particularly from chemotherapy, radiation therapy, and immunotherapy. The current literature found changes in the diversity and abundance of the skin microbiome during cancer treatments such as radiation therapy, including lower diversity of the skin microbiome, an increased *Proteobacteria/Firmicutes* ratio, and a higher abundance of pathogenic *Staphylococcus aureus*. These changes may be associated with the development and severity of treatment-related skin toxicities, such as acute radiation dermatitis, hand-foot syndrome in chemotherapy, and immunotherapy-induced rash. Several clinical guidelines have issued potential interventions (e.g., use of topical corticosteroids, phototherapy, and non-pharmaceutical skin care products) to prevent and treat skin toxicities. The effectiveness of these promising interventions in alleviating treatment-related skin toxicities should be further tested among cancer patients.

## Introduction

The human skin microbiome is composed of millions of bacteria, fungi, archaea, and viruses, which function cooperatively to protect against invasive pathogens, acquire immunity, and metabolize natural products (1–3). The skin microbiome plays a crucial role in balancing human immunological and physiological functions; disruption to the balance between skin commensals and pathogens can lead to the development of skin diseases with varying severity (4, 5). Changes in human health conditions (e.g., chronic illness, use of antibiotics, and compromised immune status) can alter the skin microbiome (1, 6). The composition of the skin microbiome is highly dependent on the physiology of the skin site, which is characterized by the microenvironment, distinguished as sebaceous, oily, moist, or dry (1, 2). Dermatological research has provided emerging insights on the composition of skin microbiome based on these sites: sebaceous sites (e.g., glabella, alar crease, back, and face) are dominated by lipophilic Cutibacterium species; moist areas (e.g., umbilicus, gluteal crease, and plantar heel) are dominated by Staphylococcus (S.) and Corynebacterium, and Cutibacterium (C.); Corynebacterium, and various Streptococcus strains have been discovered in dry areas (e.g., buttock, volar forearm, and hypothenar palm) (1, 5).

Recent research has been conducted to understand the relationship of the human gut microbiome (7–9) and vaginal microbiome (10–12) with human health and disease, including its importance as a biomarker of cancer diagnosis, treatment toxicities, and prognosis (1). However, less is known about the relationship of the skin microbiome with disease, such as cancer and cancer treatment-related toxicities. Due to the linkages between processes of inflammation and skin microbes, as well as discoveries on non-oncogenic viral interference linked to reduced cancer risks, new theories have emerged about the role of a dysbiotic skin microbiome in cancer development (13, 14), cancer treatments, and oncologic outcomes (15). Sherwani and colleagues demonstrated the effect of skin cancer on the human skin microbiome, indicating that the skin microbiome of skin cancer patients was less diverse than that of healthy controls (15). Additionally, the microbial taxa thriving within the tumor environment have been found to be tumor-specific, and they direct regulators of cancer initiation, progression, and response to chemotherapy and immunotherapies (15, 16).

Cancer treatment including chemotherapy, radiation therapy [RT], and immunotherapy have significantly improved cancer survival, but treatment-related skin toxicities are common (6). Skin toxicities differ in severity as reported in various treatments (17). Cancer treatment-related skin toxicities can be distressing as skin toxicities can alter one’s appearance and serve as a constant reminder of their ailment. Toxicities affecting quality of life (QOL) can limit patients’ daily functionality, force changes in their therapy schedules, and even lead to treatment termination (17). With RT, radiation dermatitis is a common problem affecting up to 90% of patients with RT (18–20); the severity of radiation dermatitis ranges from mild erythema to moist desquamation and can manifest as dramatic acute skin reactions (18, 21) and/or chronic skin alterations that might have a significant negative impact on patients’ QOL (18, 22, 23). Commonly reported skin toxicities include epidermal growth factor receptor inhibitor (EGFRI) rash, hand-foot skin reaction, hand-foot syndrome or palmar-plantar erythroderma, and chemotherapy-induced alopecia (17, 24).

Few studies have characterized the skin microbiome in cancer patients receiving oncologic therapy. In this mini-review, we reviewed current literature on the skin microbiome and its impact on cancer treatment-related skin toxicities. Specifically, this study reviewed: 1) cancer treatment-related skin toxicities; 2) the skin microbiome and its potential role in treatment-related skin toxicities; and 3) potential interventions for preventing and treating skin toxicities among patients with cancer. We hypothesize a decreasing diversity of the skin microbiome and an increasing pathogenic skin microbes (e.g., S. aureus) with the emergence of cancer and employment of some form of cancer therapy. Additionally, we hypothesize that chronic inflammation plays a promising mechanistic role in the skin microbiome-related treatment toxicities.

## Skin Microbiome in Cancer

Advances in microbial research have illuminated understanding of immune and inflammatory pathways in the tumor microenvironment, as well as pathogenesis and cancer progression (25). Given that chronic inflammation is known to create a pro-cancer environment and microbial dysbiosis is associated with mechanisms of inflammation, the abundance of certain microbes is linked to the development of specific cancer types, including skin cancer. Current literature has primarily focused on preclinical models (e.g., piglets and mice). A study comparing the composition and diversity of microbiota in healthy skin vs. melanoma in a pig-model found that *Fusobacterium nucleatum* promoted proliferation, binding to tumors to prevent immune cell attack and inhibit natural killer cell cytotoxicity (26, 27). More evidence showed that *Lactobacillus*, *Clostridium sensu stricto 1* and *Corynebacterium 1* were primarily dominated genera in the healthy skin, while *Fusobacterium*, *Trueperella*, *Staphylococcus*, *Streptococcus*, and *Bacteroides* were discriminately abundant in melanoma tissue (28). Healthy skin is primarily characterized with *Firmicutes*, in which *Staphylococcus*, *Streptococcus*, and *Lactobacillus* are dominant (26). Although the mechanism of bacteria-inducing tumor proliferation is not well understood, there are several contributing processes, such as the production of toxins and inflammation, which leads to DNA damage and induce a pro-inflammatory environment in the skin (25, 29–32).

Limited but promising clinical work similarly investigated the role of skin microbiome in cancer development. Voigt et al. characterized the skin microbiome in squamous cell carcinoma (SCC), its precursor, actinic keratoses as compared to healthy controls (33). Voigt et al. discovered *Cutibacterium* to be associated with healthy skin, while *Staphylococcus* was associated with actinic keratoses and SCC. Considering the antagonistic properties of *C. acnes* and *S. aureus*, researchers hypothesized that malignant tissue’s loss of its sebaceous properties may prevent growth of *C. acnes*, promoting a pro-inflammatory environment susceptible to *S. aureus* colonization (33, 34). Moreover, *Corynebacterium* genus was found associated with patients with advanced (stage III/IV) melanoma, in which IL-17 promotes the proliferation of melanoma cells through upregulation of IL-6 and signal transducer and activator of transcription 3 (35, 36). Besides skin cancer, recent preliminary work showed a high abundance of some skin *Staphylococcus* species linked with breast cancer and metastases, in which microbial transfer to underlying tissue is proposed, including retrograde transfer *via* ductal systems, skin barrier breakdown, and migration through nipple-aspirate fluid (37). As a summary, skin morphology is significantly changed during carcinogenesis and consequently the microbial communities are altered, inhabiting potentially pro-tumorigenic microbes (29). To fully evaluate the skin microbiome, we encourage future researchers to further confirm alterations in microbial profiles across the continuum of cancer care trajectory.

## Cancer Treatment-Related Skin Toxicities

### Chemotherapy

Chemotherapy-related toxicities frequently occur from systemic damage of bone marrow, hair follicles, mouth, digestive tract, and reproductive system (38, 39). Pyrimidine antagonists and anthracycline chemotherapy agents interfere with synthesis of biological molecules and ultimately block cell division, resulting in a variety of skin toxicities, including hand-foot syndrome (HFS) and chemotherapy-induced alopecia (17, 40, 41). HFS is a well-studied cutaneous adverse reaction of chemotherapy agents, such as capecitabine, 5-fluorouracil, docetaxel, and pegylated liposomal doxorubicin. HFS is not typically life-threatening; however, debilitating discomfort of the palms and soles can significantly affect patient’s QOL as well as impact treatment compliance (40). In extremely severe cases, HFS has shown to lead to tissue necrosis, requiring amputation; immunocompromised patients are also at risk for infection, making patients prone to bacterial sepsis, which could be fatal (17, 41). Chemotherapy-induced alopecia affects about 65% of patients receiving cytotoxic drugs and has a broad range of incidence depending on the therapeutic agent, dosage, administration, and other patient-related factors, such as age, comorbidities, nutritional and hormonal status (42, 43). Alopecia often raises negative attitudes towards body image and self-esteem, as it is seen as a stigmatizing reminder of patients’ disease (17, 44). Some patients even choose to forgo physician’s recommendations because of alopecia, and its impact on patients’ QOL is greatly underestimated by the medical community. Interviews of women being treated for early-stage breast cancer found most of them were greatly troubled by their appearance (17), despite alopecia being mostly reversible after treatment completion with possible complications in color, texture, and complete regrowth (44).

### Radiation Therapy

RT is part of definitive treatment for many cancers, but it can cause acute and late toxicities to healthy tissue (45, 46). An inflammatory response occurs in the initial period of RT, caused by pro-inflammatory cytokines (e.g., interleukin [IL]-1, IL-3, IL-5, IL-6, IL-8, tumor necrosis factor [TNF]-α) (47). These factors create a local inflammatory response leading to skin tissue damage and loss of protective barriers (48). Using mice models, Janko et al. found that mice lacking IL-1 or the IL-1 receptor developed less inflammation and suffered lower levels of radiation dermatitis (48). These findings signify the important role of IL-1 in the development of RT-induced skin toxicities, indicating that cytokine pathways (e.g., IL-1) have potential for precisely targeted therapy, especially considering their previous approvals for use in humans to block this cytokine (49, 50).

Acute radiation dermatitis includes symptoms, such as mild erythema and desquamation, ranging from dry desquamation to severe, confluent moist desquamation. When the skin basal cells are destroyed, the balance of normal cell production is disrupted. As the total RT dose accumulates with treatment, the protective skin barrier becomes impaired and dysfunctional (51). The human skin consists of trillions of rapidly proliferating and maturing cells, thus the skin experiences high levels of radiosensitivity and can have dramatic toxicities. While the exact mechanism is unknown, some proposed theories include basal cell proliferation, endothelial cell damage, alterations in membrane permeability, and release of inflammatory cytokines (51–56). A supportive skin care regimen is important in maintaining the integrity of the epidermal barrier, and thus treating irritating symptoms, such as desquamation, xerosis, erythema, pruritus, and hyperpigmentation (51).

### Immunotherapy

Immunotherapy targets immune checkpoint pathways through a class of negative key regulators of T cell activation: cytotoxic T-lymphocyte-associated protein-4 (CTLA-4) and programmed cell death protein-1 (PD-1). The primary biological function for these immune checkpoint inhibitors (ICIs) is to induce a pro-inflammatory state in the tumor microenvironment, modulating the cellular immune response to specific tumor antigens and killing tumor cells. However, lack of specificity of immune activation and mediation of ICIs has led to several different skin immune-related adverse events (irAEs). Specifically, patients with advanced melanoma, who were treated with anti-CTLA-4 (ipilimumab) or anti-PD-1 (nivolumab and pembrolizumab), reported immune side effects, such as skin rash, pruritus, and vitiligo early on during ICIs treatment, approximately 2-8 weeks after initiation. IrAEs can be even more common during combined therapies (anti-CTLA-4 and anti-PD-1) (57).

Researchers have defined irAEs as different than damage to single dermatologic units. The most prevalent irCAE is maculopapular rash, which manifests as multiple pruritic erythematous macules and papules on the trunk and extensor surfaces of the extremities. PD-1 inhibitors are known to cause a lichenoid eruption, which is characterized by papules, pruritic hypertrophic plaques, or patches (57).

## Skin Microbiome in Skin Toxicities

Most skin diseases or infections are associated with skin microbiome dysbiosis, a term that describes a disruption or imbalance in microbiota homeostasis (1, 4, 5). When the stability of the skin microbiome is threatened, the individual’s skin sites can become populated with pathogenic bacteria, such as *S. aureus*, presenting significant risks of infection (1, 2, 58). The skin microbiome serves as a modulator between symbiotic commensals that provide a wide variety of skin niches to protect against invasion of pathogenic microorganisms. Chemotherapy-induced damage to skin and hair follicle cells alters the skin’s microbial environment. In particularly, a significant increase in microbial diversity (i.e., Shannon) and decrease of *S. aureus* proportion were observed with eczema treatment by topical corticosteroid (58). A knowledge gap still exists regarding the biological mechanism of skin microbiome dysbiosis leading to chemotherapy-induced skin toxicities, such as alopecia and hand-foot syndrome. Understanding the skin microbiome and its associations with chemotherapy-related skin toxicities can aid in the development of strategic planning and therapeutic interventions that increase patients’ QOL.

Characterization of the skin microbiome profiles associated with RT-induced dermatitis could help elucidate the mechanisms of pro-inflammatory cytokines and possibly identify targets to decrease RT-induced skin toxicities. However, the relationship between the skin microbiome and immune system is not well-understood. In contrast, atopic dermatitis, or eczema, is a chronic allergic skin disease that has been extensively studied regarding its relationship to the skin microbiome (56, 59). With flareups of atopic dermatitis, healthy skin microbial flora approach a diseased state as defective genes lead to Th2-mediated immunological disruptions in the skin barrier, thereby accelerating susceptibility to infection (56, 60). Physical irritation, such as excoriation of dry and inflamed skin, can further exacerbate microbiome dysbiosis (59). Microbial homeostasis is mediated by Th2 cytokines, which suppress keratinocyte induction of antimicrobial peptides, including human beta-defensin-3 and cathelicidins that prevent colonization of pathogenic organisms (61–63). Increased rates of pathogenic *S. aureus* and decreased diversity of other microorganisms are consistently reported in atopic dermatitis lesions in comparison to the healthy skin (64–67). As reported by Ramadan and colleagues (68), cancer patients with RT-induced dermatitis had significant reduction in bacterial diversity (Shannon, Chao1, and observed species) comparison to healthy controls. The delayed recovery or tendency toward the permanence of RT-induced dermatitis were associated with a raised *Proteobacteria/Firmicutes* ratio as well as the dermotype with overrepresentation of *Pseudomonas, Staphylococcus*, and *Stenotrophomonas*. With limited evidence, these findings need to be further confirmed in patients with cancer receiving RT.

The diversity and stability of the skin microbiome differs across locations and is of particular interest to understanding regulation of the immune response, as well as the progression of chronic skin disease, such as atopic dermatitis and psoriasis. Research has proposed that a lack of cutaneous microbial diversity and greater density of *S. aureus* communities are associated with increase inflammation and disease pathogenesis (65–67, 69, 70). Psoriasis is a chronic inflammatory skin disease that appears as raised, scaly, erythematous lesions, known to be triggered by disruptions in the immune system (71, 72). While previous research has shown a decrease in commensal diversity in psoriasis, there have been conflicting reports of the level of abundance in *Firmicutes* and *Actinobacteria*, as well as in the major species, including *Corynebacterium, Cutibacterium*, *Staphylococcus*, and *Streptococcus* (73–76). These studies demonstrated how the nature of the skin microbiome stimulates the innate and adaptive immune response. However, the pathways and microbial communities significant to immunotherapy-related skin toxicities are still unknown.

A healthy skin microbiome protects against pathogenic organisms, whereas disruptions in the microenvironment can introduce skin irritations, including acute dermatitis and psoriasis, as well as skin toxicities caused by cancer treatment. Future studies will likely utilize whole genome sequencing to approach the direct mechanism between microbes and the host to evaluate therapeutic targeting potentials of the skin microbiome in irritated skin. By assessing the key patterns in microbial dysbiosis, we can address specific QOL concerns with earlier diagnosis and improved treatment strategies.

## Clinical Guidelines and Interventions for Skin Toxicities

**Figure 1:**
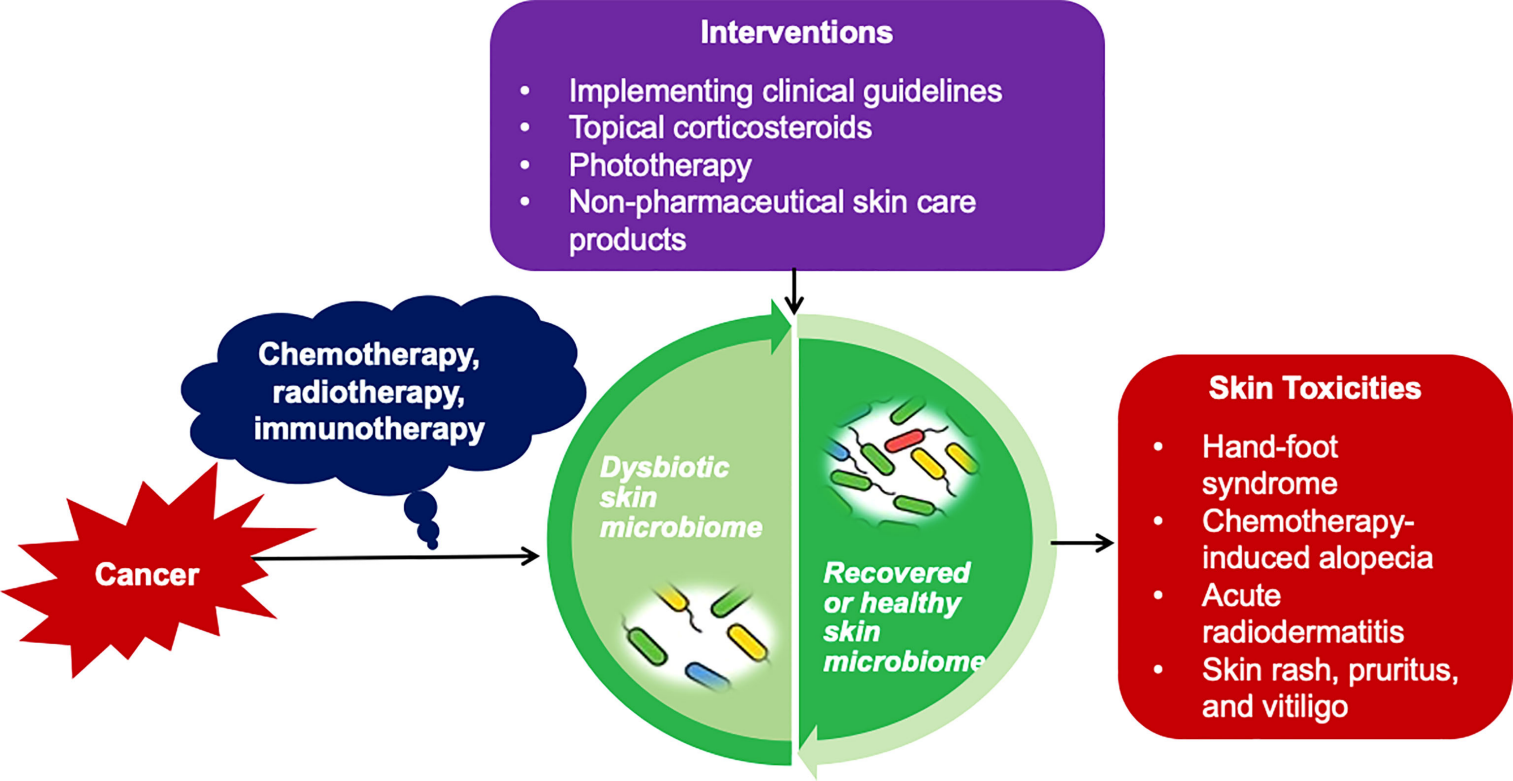
Impact of Skin Microbiome on Cancer Treatment-related Skin Toxicities and Potential Interventions. Based on current knowledge of the bacterial microbiome in cancer treatment-related toxicities, implementing clinical guidelines, topical corticosteroids, phototherapy, and non-pharmaceutical skin care products are suggested approaches for the early prevention and management of cancer treatment-related skin toxicities via adjusting the skin microbiome, and eventually improve patients' quality of life.

Management of cancer treatment-related skin toxicities composes several different facets, including patient education, early prevention, dosage regulation, and symptom assessment and management. Patient’s QOL guidelines have been developed to determine the necessity and scale of treatment suspension, dose modification, and treatment options for skin toxicities. Incorporating the management guidelines related to skin toxicities into clinical practice and testing promising interventions can potentially address the skin toxicities early, reduce patients’ skin symptom burden, and improve patients’ QOL (**Figure 1**) (17).

### Clinical Guidelines

Clinical practice guidelines have been developed to support patients and clinicians in decisions regarding management of treatment-related skin toxicities. The Multinational Association of Supportive Care in Cancer (MASCC) in 2011 published practice guidelines for the prevention and treatment of EGFRI-associated dermatologic toxicities (77). Recently, the Oncology Nursing Society (ONS) Guidelines™ detailed specific recommendations on early prevention and treatment of EGFRI rash, hand-foot skin reaction, HFS, and chemotherapy-induced alopecia (17). For the prevention of EGFRI rash, both ONS and MASCC guidelines recommended topical hydrocortisone 1% cream with moisturizer, twice-daily sunscreen application, and oral antibiotics; to treat the EGFRI rash, both guidelines recommend topical steroids and oral antibiotics (17, 77). Both guidelines further recommended topical minoxidil, a class of drugs called vasodilators, for chemotherapy-induced alopecia (17, 77).

### Topical Corticosteroids

For chemotherapy-induced skin reactions, topical steroids in combination with vasoconstrictors are effective in preventing permanent alopecia by inhibiting damage to hair follicle stem cells (58, 61). Acute RT-induced skin reactions are inflammatory reactions that are often treated with topical corticosteroids to vasoconstrict blood vessels, reduce capillary permeability, and inhibit leukocyte proliferation (61). Mometasone furoate is a highly potent corticosteroid that has been shown to significant decrease the pro-inflammatory mediators during breast RT and decrease acute radiation dermatitis (61).

### Treatment for irAEs From Immunotherapy

With immunotherapy, treatment options for irAEs are based on dose and grade of skin toxicities. For example, a low-grade maculopapular rash can be managed with mid- to high-potency topical corticosteroids; more severe skin reactions require systemic corticosteroids and can even lead to early ICI termination (44). One study showed that a full recovery from Sweet’s syndrome (i.e., a class of neutrophilic dermatoses) was achieved *via* oral and intravenous corticosteroids. Continuation of management of high doses of steroids and other immune mediators for irAEs may be hampered as it often counters the therapeutic potency of ICIs. To avoid discontinuation of immunotherapy, other agents to symptomatically treat irAEs are often introduced, including oral retinoids with phototherapy. Other third line therapy options include cyclosporine, methotrexate, and anti-TNF-α. Acitretin, apremilast, and methotrexate are recommended prior to systemic corticosteroids, and biological drugs, including anti-TNF-α, anti-IL-17, and anti-IL-23, can be effective in treating psoriatic lesions and have been used to treat psoriasiform rash from ICIs (51).

### Phototherapy

Atopic dermatitis and psoriasis are chronic inflammatory skin diseases that closely intertwine with changes in the skin microbiome (58–62). *S. aureus* plays an important role in the disease’s development, and its colonization of the skin microbiome is closely related to disease severity (58, 59). Narrowband ultraviolet B radiation has become a common treatment option for patients with differing levels of these adverse skin reactions because it signals the release of antimicrobial peptides, thereby reducing the *S. aureus* count and ultimately affecting the innate immune system (74, 76). Narrowband ultraviolet B phototherapy paired with corticosteroids could positively affect the skin microbiome by increasing microbial diversity and decreasing the proportion of S. aureus. Utilizing skin swabs and high-throughput sequencing of 16S ribosomal RNA genes could help further our understanding of the skin microbiome and skin toxicities from cancer treatment.

### Non-pharmaceutical Skin Care Products

Commercially available, non-pharmaceutical skin care products have been used to reduce radiation dermatitis. Berger et al. reported that patients with breast cancer undergoing RT who were provided with a kit of skin products, including thermal water spray, emollient, cleanser, wound healing cream, and sunscreen, had significantly fewer skin reactions compared to patients who did not receive the kit (51). Proactive skincare is recommended by physicians for patients undergoing RT as these products aid in minimizing skin reactions by maintaining the epidermal barrier and possibly stabilizing microbial homeostasis. With the use of prophylactic skin care, the microenvironment is kept intact and better suited for a healthy skin microbiome, resulting in less severe skin toxicities among patients with cancer (51).

## Conclusions

The human skin microbiome has proven to have a profound relationship with the innate and adaptive host immune system in particular skin disorders, such as atopic dermatitis and psoriasis. The skin microbial communities help maintain the skin microenvironment through stimulation and inhibition of the gene expression of host-produced immune factors and pro-inflammatory cytokines. Cancer treatments including chemotherapy, RT, and immunotherapy can cause prominent adverse skin toxicities, such as dermatitis, rash, alopecia, among other irritating skin reactions. Although few studies have characterized the skin microbial profiles associated with skin toxicities, some extrapolation from pro-inflammatory skin disorders like atopic dermatitis and psoriasis can be pursued. Well-developed clinical studies investigating the role of the skin microbiome in cancer treatment-related skin toxicities are needed.

## Author Contributions

Conception of the study (BR, JL, ZB, and JB), developing the search strategy (BR and JB), conducting the literature search and summary (BR), drafting the article (BR), editing the article (JL, ZB, and JB), and funding acquisition (JL and JB). All authors contributed to the article and approved the submitted version.

## Funding

This work was partially supported by the Emory University Woodruff Health Science Center Synergy Award (PIs: J Bai and J Lin) and National Institutes of Health/National Institute of Nursing Research (1K99NR017897-01 and 4R00NR017897-03, PI: J Bai).

## Conflict of Interest

The authors declare that the research was conducted in the absence of any commercial or financial relationships that could be construed as a potential conflict of interest.

## Publisher’s Note

All claims expressed in this article are solely those of the authors and do not necessarily represent those of their affiliated organizations, or those of the publisher, the editors and the reviewers. Any product that may be evaluated in this article, or claim that may be made by its manufacturer, is not guaranteed or endorsed by the publisher.

## Abbreviations

Cutibacterium acnes C. acnes, CTLA-4, cytotoxic T-lymphocyte-associated protein-4; EGFRI, epidermal growth factor receptor inhibitor; HFS, hand-foot syndrome; irAEs, immune-related adverse events; IL, Interleukin; ICIs, immune checkpoint inhibitors; PD-1, programmed cell death protein-1; QOL, quality of life; RT, radiation therapy;Staphylococcus aureus, S. aureus;

SCC, squamous cell carcinoma; TNF, tumor necrosis factor.
